# DW-F5: A novel formulation against malignant melanoma from *Wrightia tinctoria*

**DOI:** 10.1038/srep11107

**Published:** 2015-06-10

**Authors:** Jayesh Antony, Minakshi Saikia, Vinod. V, Lekshmi. R. Nath, Mohana Rao Katiki, M.S.R. Murty, Anju Paul, Shabna A, Harsha Chandran, Sophia Margaret Joseph, Nishanth Kumar. S, Elizabeth Jayex Panakkal, Sriramya I. V, Sridivya I. V, Sophia Ran, Sankar S, Easwary Rajan, Ruby John Anto

**Affiliations:** 1 Division of Cancer Research, Rajiv Gandhi Centre for Biotechnology, Thiruvananthapuram 695 014, Kerala, India; 2 Medicinal Chemistry and Pharmacology Division, Indian Institute of Chemical Technology, Hyderabad 500007, India; 3 Agroprocessing and Natural Products Division, National Institute for Interdisciplinary Science and Technology (NIIST), Council of Scientific and Industrial Research (CSIR), Thiruvanathapuram 695 019, Kerala, India; 4 Department of Medical Microbiology, Immunology and Cell Biology, Southern Illinois University-School of Medicine, P.O. Box 19626, Springfield, Illinois, USA; 5 Department of Pathology, Government Medical College, Thiruvananthapuram 695 011, Kerala, India; 6 Department of Chemistry, Sree Kerala Varma College, Thrissur 680011, Kerala, India

## Abstract

*Wrightia tinctoria* is a constituent of several ayurvedic preparations against skin disorders including psoriasis and herpes, though not yet has been explored for anticancer potential. Herein, for the first time, we report the significant anticancer properties of a semi-purified fraction, DW-F5, from the dichloromethane extract of *W. tinctoria* leaves against malignant melanoma. DW-F5 exhibited anti-melanoma activities, preventing metastasis and angiogenesis in NOD-SCID mice, while being non-toxic *in vivo*. The major pathways in melanoma signaling mediated through BRAF, WNT/β-catenin and Akt-NF-κB converging in MITF-M, the master regulator of melanomagenesis, were inhibited by DW-F5, leading to complete abolition of MITF-M. Purification of DW-F5 led to the isolation of two cytotoxic components, one being tryptanthrin and the other being an unidentified aliphatic fraction. The overall study predicts *Wrightia tinctoria* as a candidate plant to be further explored for anticancer properties and DW-F5 as a forthcoming drug formulation to be evaluated as a chemotherapeutic agent against malignant melanoma.

Scientific validation of ethno botanical knowledge may lead to the discovery of specific and non-toxic anti-cancer formulations. Here we report the anti-melanoma efficacy of a semi- purified fraction (DW-F5) isolated from *Wrightia tinctoria* (Roxb.) R.Br., a small medicinal tree native to India and Burma and is known in Siddha and Ayurvedic system of Indian traditional medicine as a cure for various skin related ailments. Different parts of this plant have been accredited for alleviating diseases such as psoriasis and herpes^1^,^2^ and for the preparation of hair oils used for treating various scalp and skin disorders^1^,^3^. Additionally, the antioxidant, antiviral, antiulcer and wound healing properties of *Wrightia tinctoria* have also been well documented^4^,^5^,^6^,^7^. However no scientific study has been reported on the anti-cancer activity of this plant except some anecdotal information regarding its use as a traditional remedy for curing lung and breast cancer, which is not validated through scrupulous scientific experiments or systematic studies^8^.

Melanoma is an aggressive form of human cancer arising from the malignant transformation of the pigmented cells of skin. Constitutive activation of MAPK [Mitogen-activated protein kinase] due to the gain-of-function mutation of its up-stream regulator BRAF(serine/threonine-protein kinase B-Raf)^12^ and survival signals activated due to the over-expression of oncogenic transcriptional factors such as MITF-M [Microphthalmia-associated transcription factor], β-catenin and Brn-2 (POU domain, class 3, transcription factor 2)^11^,^22^,^23^,^24^,^25^,^28^, are crucial factors known to play significant role in melanoma progression. These signaling malfunctions, in solo or in combination, could create a cellular environment conducive for melanoma propagation leading to its aggressiveness. Despite extensive research, the chemotherapeutic options for melanoma cure remain dismal due to the insensitivity of melanoma cells to conventional anti-cancer drugs. This study demonstrates the promising effects of DW-F5 in blocking the melanoma growth both *in vitro* and *in vivo* along with its efficacy in modulating signals known to play a crux role in melanoma progression.

## Results

### Dichloromethane extract of *Wrightia tinctoria* leaves induces maximum cytotoxicity towards melanoma cells

Human cancer cells of different origin were treated with hexane (HEWL-Hexane Extract of *Wrightia* Leaves), dichloromethane (DEWL- Dichloromethane Extract of *Wrightia* Leaves), ethyl acetate (EEWL- Ethyl acetate Extract of *Wrightia* Leaves) and methanol (MEWL- Methanol Extract of *Wrightia* Leaves) extracts (SI methods), which were obtained by a polarity graded successive extraction of the leaves. The cytotoxic effect of these extracts was evaluated using MTT assay. The results showed that DEWL and EEWL induced considerable cytotoxicity towards skin cancer cells (IC50-20 μg/ml and 36 μg/ml respectively), compared to HEWL and MEWL (IC50-100-250 μg/ml) (Supplementary Fig. 1a–d). As DEWL was the most active extract (Patent Application No. 2743/CHE/2010), it was selected for further purification, and its cytotoxicity was tested in skin cancer cell lines A375, A431 and SK-MEL-2 (SI methods), which confirmed A375 as the most sensitive cell line (Supplementary Fig. 2) and hence it was selected for subsequent studies.

### Bioactivity guided fractionation of DEWL leads to the isolation of DW-F5

Successive silica gel column chromatography of DEWL led to the isolation of DW-F5 [DCM *Wrightia*-Fraction 5] (SI methods), which exhibits a drastic increase in cytotoxicity (IC50-8.8 μg/ml) (Supplementary Fig. 3). IR spectrum of DW-F5 exhibited peaks indicative of two carbonyl groups, and ^1^H NMR spectrum (Supplementary Fig. 4a,b) displayed peaks due to aromatic protons, demonstrating that the fraction contains an aromatic compound with carbonyl functionality. The spectrum also contains peaks in the aliphatic region which may be indicative of an impurity or another molecule contributing to the activity of the fraction.

### Toxicological evaluation of DW-F5

**Figure 1:**
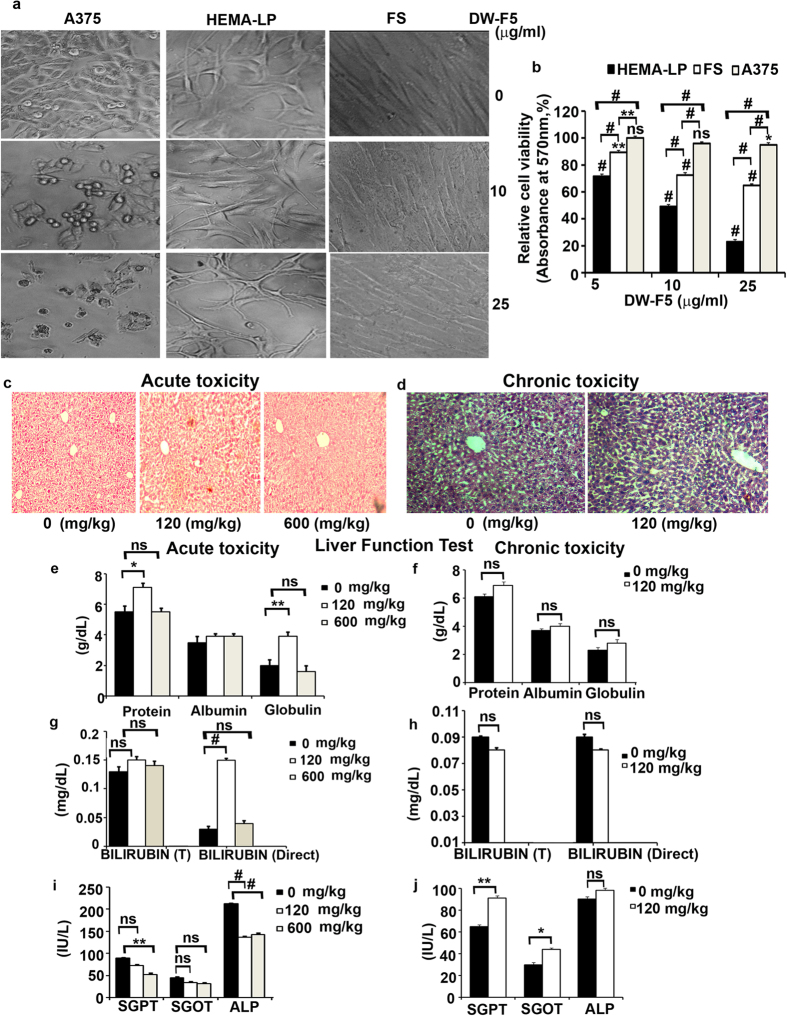
DW-F5 does not induce pharmacological toxicity. **(a,b)** DW-F5 induces significant cytotoxicity in the melanoma cell line, A375, while normal skin fibroblast (FS) and epithelial cells (HEMa-LP) are unaffected as determined by microscopic observation and MTT assay. **(c,d)** H&E stained liver tissues of mice treated with or without DW-F5 for a period of 7 days and 3 months. **(e,f)** The serum level of total proteins, albumin and globulin in DW-F5 treated mice for a period of 7 days and 3 months is represented graphically. **(g,h)** Graphical representation of the serum level of total and direct bilirubin in DW-F5 treated mice for a period of 7 days and 3 months. **(i,j)** The activity of toxicological markers such as serum alkaline phosphatase (ALP), serum glutamic oxaloacetic transaminase (SGOT) and Serum glutamic pyruvic transaminase (SGPT) in DW-F5 treated mice for a period of 7 days and 3 months is graphically represented. Though there was a statistically significant deviation in the values of some toxicological parameters between the control and treated groups, the varied values were within the normal range. Data represent three independent sets of experiments. The error bars represent ± S.D. ANOVA (for MTT assay) or Student’s t test (for toxicological studies) was used for statistical comparison among different groups. * P ≤ 0.05; ** P ≤ 0.01; # P ≤ 0.001, ns non significant.

While the fraction induced significant cytotoxicity in melanoma cells (IC50-8.8 μg/ml) (Fig. 1a,b), it was non-toxic to normal skin fibroblasts and melanocytes even at double the concentration (Fig. 1a,b) that almost completely killed the cancer cells, indicating its biological safety. Pilot studies revealed that a dose of 120 mg/Kg of DW-F5 in NOD-SCID [Non Obese Diabetic-Severe Combined Immuno Deficiency ] mice displayed significant inhibition of tumour growth in 90% of the animals studied without mortality. We confirmed the pharmacological safety of this dose by including a five times higher concentration (600 mg/Kg) in the acute and chronic toxicity studies (SI Methods)^9^. None of the doses induced liver toxicity except a mild dilation of sinusoids in the group treated with 600 mg/Kg of DW-F5, which indicates mild cholestasis (Fig. 1c,d). No necro inflammatory reactions, fatty changes or obvious/irreversible liver damage were observed in any of these tissues. Though some of the biochemical parameters reveal significant deviation in response to DW-F5 administration during toxicity evaluation, none of it exceeds beyond the normal range (Fig. 1e–j), establishing the pharmacological safety of DW-F5, *in vivo*. Hence, 120mg/Kg dose was selected for evaluating the anticancer potential.

### Evaluation of DW-F5 for apoptotic and anticlonogenic activity

**Figure 2:**
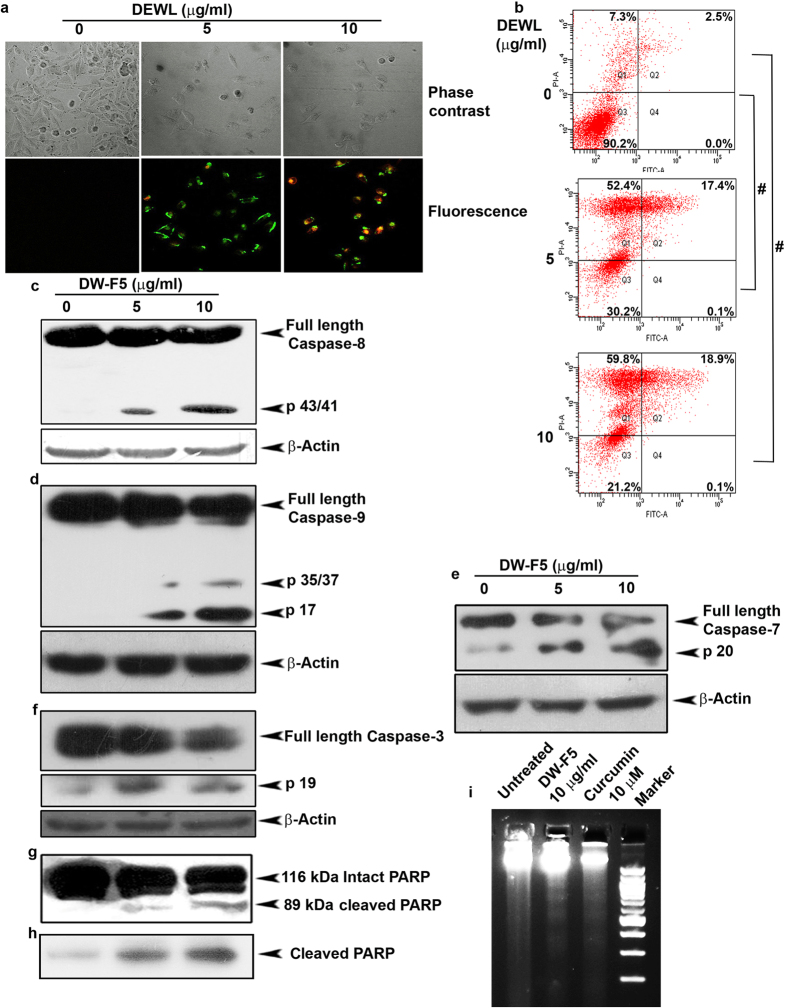
DW-F5 induces apoptosis in melanoma cells. **(a)** Cells were treated with DW-F5 for 24 h, washed with PBS and stained with annexin V–FITC/propidium iodide mixture, and photomicrographed (40X). **(b)** Cells were exposed to DW-F5 for 48 h, stained with fluorescein isothiocyanate (FITC)-conjugated Annexin V and propidium iodide and subjected to flow cytometry. The population of Annexin/ PI-positive cells in the top right and bottom right quadrants represents the total percentage of apoptotic cells. **(c–g)** Western blots showing DW-F5-induced caspase activation and PARP cleavage in A375 cells. **(h)** Western blot showing cleaved PARP in A375 cells after DW**-**F5 treatment **(i)** Agarose gel showing the effect of DW-F5 on internucleosomal DNA fragmentation in A375 cells. All experiments were repeated thrice. Statistical significance was analysed using Student’s t test. # P ≤ 0.001.

To test whether DW-F5 induces apoptosis in A375 cells, we compared the extent of Annexin-PI [Propidium Iodide] positivity in both DW-F5-treated and control cells, by confocal microscopy as well as FACS analysis (SI Methods). Annexin V has a strong affinity towards phosphatidyl serine, which is externalized due to membrane flip-flop, an early event of apoptotic program. A dose dependent increase in the number of Annexin-PI positive cells was observed in DW-F5 treated cells, thus authenticating apoptosis (Fig. 2a). Corroborating this observation, flow cytometric analysis of DW-F5 treated cells stained with fluorescein isothiocyanate conjugated Annexin V and propidium iodide displayed a very significant increase in the percentage of apoptotic cells (Fig. 2b). The involvement of classical caspase cascade leading to DNA fragmentation was also investigated in DW-F5-induced apoptosis. The fraction brought about a dose dependent cleavage of both initiator (Fig. 2c,d) and effector caspases (Fig. 2e,f) and their downstream substrate, PARP [Poly ADP ribose polymerase] (Fig. 2g,h) which resulted in fragmentation of DNA (Fig. 2i).

DAPI staining was also performed to substantiate apoptosis in DW-F5 treated A375 cells (SI methods), which exhibited momentous increase in the number of fragmented or condensed chromatin, as assessed by enhancement in fluorescence intensity (Supplementary Fig. 5a).

The efficacy of the fraction to inhibit the proliferative capacity of the cells was determined by clonogenic assay (SI methods). Compared to the untreated cells, DW-F5 induced a dose dependent and highly significant reduction in the number and size of A375 clones (Supplementary Fig. 5b,c).

### DW-F5 down-regulates survival signals prevalent in melanoma

**Figure 3:**
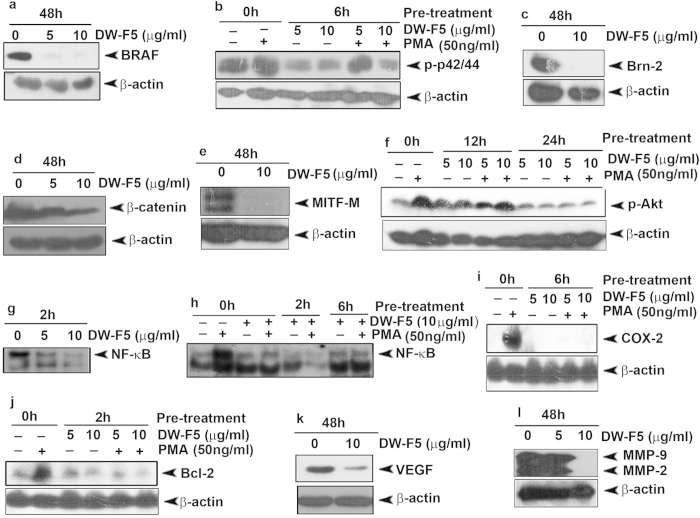
DW-F5 down-regulates activation of survival signals. (**a)** DW-F5 drastically inhibits the BRAF expression in A375 cells. **(b)** DW-F5 down-regulates the constitutive and PMA-induced phosphorylation of ERK1/2. **(c-e)** DW-F5 down-regulates Brn-2, β-catenin and MITF-M**. (f)** DW-F5 down-regulates constitutive and PMA-induced phosphorylation of Akt. **(g,h)** DW-F5 down-regulates nuclear translocation of NF-кB. **(i,j)** DW-F5 down-regulates constitutive and PMA-induced up-regulation of COX-2 and Bcl-2. **(k,l)** DW-F5 down-regulates VEGF and MMPs. The details of treatment modes are provided in SI methods.

Our next attempt was to analyse the effect of DW-F5 on constitutive and PMA (phorbol-12-myristate-13-acetate)-induced expression of survival signals and transcription factors prevalent in melanoma signaling (SI methods). Mutated BRAF leads to the constitutive activation of MAPK signaling in melanoma^10^ and its inhibition leads to tumour regression^11^. DW-F5 down-regulate almost completely the oncogenic BRAF (Fig. 3a), which is reported to be mutated and activated in A375 cells^12^. We also tested whether DW-F5 is able to down-regulate the activation of ERK1/2 [Extracellular signal-regulated kinases 1/2], which is constitutively activated in A375 cells due to their mutant BRAF status 13 and observed that 6h pre-treatment with DW-F5 down- regulates both the constitutive and PMA-induced phosphorylation of ERK1/2 (Fig. 3b). BRAF mutation activates ERK1/2 which in turn up-regulates Brn-2, a molecule highly over-expressed in BRAF mutant melanoma cells, but not in melanocytes or melanoblasts 14. Interestingly, DW-F5 could completely abolish Brn-2 expression (Fig. 3c).

Since Wnt/ β-catenin signaling pathway is strongly implicated in melanoma progression, we tested whether the cytotoxic effect exerted by DW-F5 is associated with the down-regulation of key proteins involved in this pathway. A substantial down-regulation of β-catenin upon treatment of cells with DW-F5 was observed (Fig. 3d) indicating that Wnt signaling pathway may have a regulatory role in DW-F5-induced cytotoxicity. All the pathways discussed above converge into MITF-M, which is reported to be the pivotal molecule in melanoma progression^15^. Interestingly, DW-F5 completely abolished the constitutively expressed MITF-M in melanoma (Fig. 3e).

To evaluate whether the melanoma specific molecules, Brn-2 and MITF-M have crucial role in regulating DW-F5-induced cytotoxicity, we analysed the expression pattern of these molecules in response to DW-F5, in all the three skin cancer cell lines, which were initially screened for DEWL-induced cytotoxicity. While the melanoma cell lines A375 and SK-MEL-2 over-express these molecules, the non-melanoma cell line, A431 had a very feeble expression (Supplementary Fig. 6b,c), which is correlating with their cytotoxicity data (Supplementary Fig. 2). While DW-F5 abolished both the molecules in A375, it failed to completely inhibit MITF-M in SK-MEL-2 at the concentrations used (Supplementary Fig. 6b,c), though there was a substantial down-regulation, which may be the reason for the resistance of this cell line to DW-F5 compared to A375 (Supplementary Fig. 6a).

Moreover, both constitutive^16^,^17^ and PMA-induced activation of Akt [Protein kinase B (PKB)] (Fig. 3f) and NF-κB [nuclear factor kappa-light-chain-enhancer of activated B cells] (Fig. 3g,h) were also down-regulated by DW-F5 in A375 cells. Next we investigated the effect of DW-F5 on COX-2 [Cyclooxygenase-2] and Bcl-2 [B-cell lymphoma 2], which in many occasions function as down-stream targets of NF-κB, though Bcl-2 can also act independent of NF-κB^17^. Interestingly, DW-F5 treatment abolished PMA-induced up-regulation of COX-2 and Bcl-2 (Fig. 3i,j).

### DW-F5 inhibits development of melanoma tumour in NOD-SCID mice

**Figure 4:**
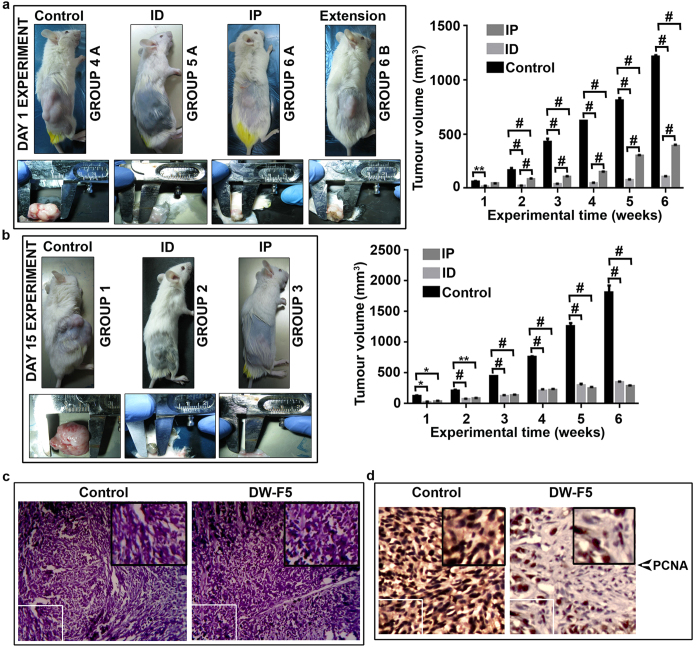
DW-F5 inhibits melanoma tumourigenesis in NOD-SCID mice. **(a)** Representative images of tumour bearing mice which received DW-F5 treatment next day after tumour implantation and the graphical representation of the tumour volume. (Day 1 group) **(b)** Representative images of mice bearing tumour which received DW-F5 treatment 15 days after tumour implantation (Day-15 group) and the graphical representation of the tumour volume. **(c)** Images of H&E stained tumour tissue of control and DW-F5 treated mice. **(d)** Figure showing the expression of PCNA in tumour tissues of control and DW-F5 treated mice. Data represent three independent sets of experiments. The error bars represent ± S.D. Statistical significance was analysed using ANOVA. * P ≤ 0.05; ** P ≤ 0.01; # P ≤ 0.001.

An orthotopic xenograft model (Supplementary Fig. 7) of melanoma was used to validate the anticancer efficacy of DW-F5 *in vivo* (SI methods & Fig. 4a,b). Surprisingly, compared to untreated (Group 4A) animals (623 mm^3^ ± 51.61), no measurable tumours were developed till the end of the experiment in group 5 and 6 animals [Group 5 & 6 A], which started receiving DW-F5, the very next day after tumour implantation [Day 1 experiment], though very small tumours (40 mm^3^ ± 7.07 for i.d. [Intradermal] & 141 ± 6.36 for i.p. [Intraperitoneal]) were observed beneath the skin after sacrificing. Some of these animals did not develop even palpable tumours, which arouse a confusion that, administration of the drug, the very next day of implantation might have killed the cells, without allowing them to form a tumour. Hence representatives of these animals [Group 6A& B] were kept for two more weeks, keeping proper controls [Group 4B], without giving DW-F5. Interestingly, tumours developed in these animals, though very small (98 ± 9.89 mm^3^ for i.d. & 382 ± 21.21 mm^3^ for i.p.) compared to that of untreated controls (1224 ± 84.85) (Fig. 4a). Moreover, intradermal administration of DW-F5 was found to be more effective, as evidenced by a significant reduction in tumour volume, when compared to that of intraperitoneal mode of administartion. On the other hand, group 2 and 3 animals that started receiving DW-F5, 15 days after tumour implantation [Day 15 experiment], developed measurable tumours, the growth of which were also significantly inhibited by DW-F5 [control:1728 ± 103.23; i.d. :348 ± 35.35 & i.p. : 271 ± 64.34] (Fig. 4b). Histopathological analysis indicated a massive destruction of cells in DW-F5 treated tissues (Fig. 4c). The drastic decrease in the expression of the cell proliferation biomarker, PCNA [Proliferating cell nuclear antigen] observed in the tumour tissues of mice treated with DW-F5 [Group 5A] as assessed by IHC analysis also correlated with the extent of tumour reduction (Fig. 4d).

**Figure 5:**
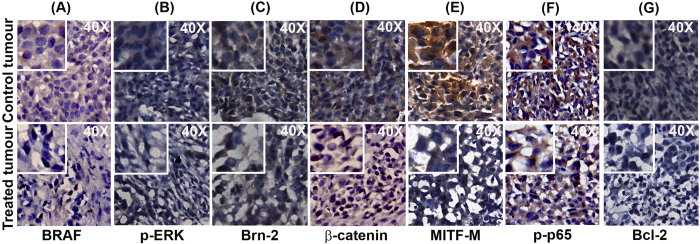
DW-F5 down-regulates key molecules crucial for melanoma survival. **(a–g)** Tumour tissues of mice treated with DW-F5 shows marked reduction in the expression of molecules such as **(a)** BRAF, **(b)** p-ERK1/2, **(c)** Brn-2, **(d)** β-catenin, **(e)** MITF-M, **(f)** p-p65 and **(g)** Bcl-2 as assessed by IHC.

Supporting the *in vitro* results (Fig. 3a), the tissue expression status of BRAF in DW-F5-treated group [Group 5A] was considerably less compared to that of untreated controls (Fig. 5a). A strong down-regulation in the BRAF-induced constitutive expression of p-ERK1/2 (Fig. 5b) was also noted in these tissues authenticating the *in vitro* results (Fig. 3b). Moreover, a complete abolition in the expression of Brn-2 (Fig. 5c), the down-stream target of both BRAF/ERK1/2 and WNT/β-catenin pathways and a drastic inhibition in the nuclear translocation of β-catenin (Fig. 5d) were also observed in the treated tissues [Group 5A], substantiating the *in vitro* results (Fig. 3c,d). These results confirm that both these pathways have a considerable role in regulating the anticancer effect of DW-F5. Most importantly, the expression of MITF-M, which is considered as the master regulator of melanoma signaling was drastically down-regulated by DW-F5 (Fig. 5e), exactly correlating with the *in vitro* data (Fig. 3e), authenticating its regulatory role.

The inhibitory effect of DW-F5 in Akt- NF-κB pathway was also illustrated in the IHC data, which exhibited complete inhibition in the nuclear translocation of p-p65, the active subunit of NF-κB and its downstream target Bcl-2 in the treated [Group 5A] tissues (Fig. 5f,g), which strongly supports the *in vitro* data (Fig. 3g,h,j).

### DW-F5 reduces the melanoma metastasis & angiogenesis in NOD-SCID mice model

**Figure 6:**
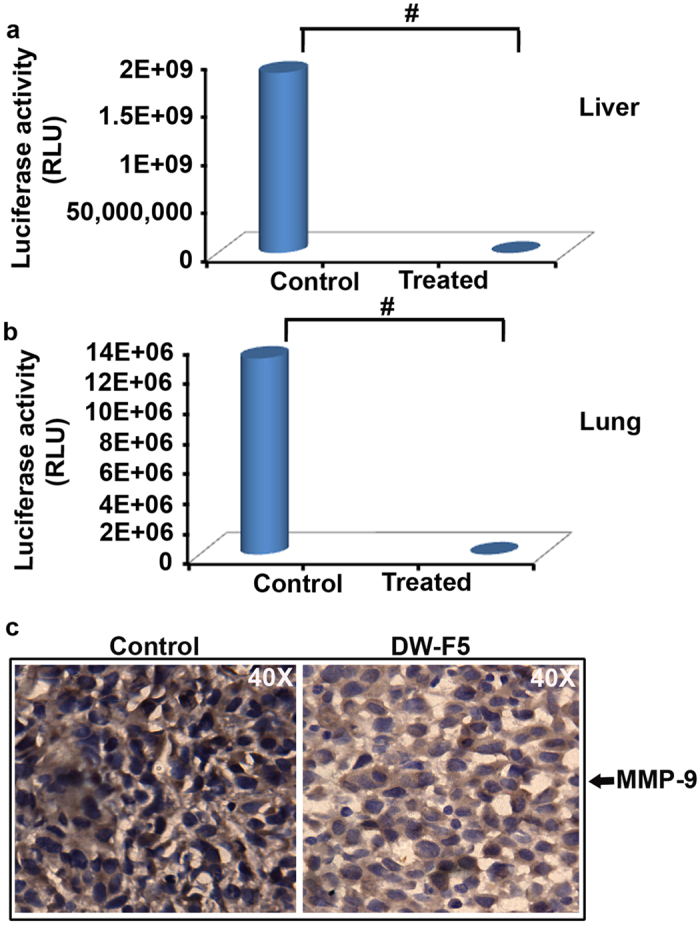
DW-F5 reduces melanoma metastasis. **(a,b)** Graph representing reduction in the metastatic spread of A375-Ren-Luc cells in liver and lungs upon DW-F5 treatment, as assessed by *in vitro* luciferase assay. **(c)** Tumour sections of mice treated with DW-F5 shows reduction in the expression of MMP-9 as assessed by IHC. Data represent three independent sets of experiments. The error bars represent ± S.D. Statistical significance was analysed by Student’s t test. # P ≤ 0.001.

To study the anti-metastatic and anti-angiogenic potential of DW-F5, A375-Ren-Luc (A375-Renilla-Luciferase) were injected intradermally to the flank region of the mice to produce orthotopic tumours (SI Methods) and were kept for two months along with DW-F5 administration and analysed for metastasis. We observed that DW-F5 treatment has significantly reduced the metastatic spread of A375-Ren-Luc cells to the prominent sites of melanoma metastasis, such as the liver and lungs by DW-F5 treatment, as assessed by *in vitro* luciferase assay (SI Methods). The relative renila luciferase activity (Rluc) was found to be more than 3 × 10^5^ times greater in the liver (P-value ≤ 0.0001) and 2.5 × 10^3^ times in the lung tissues (P-value ≤ 0.001) of the untreated animals than that of DW-F5 treated animals, demonstrating its potential as a strong inhibitor of metastasis (Fig. 6a,b). The anti-metastatic potential of DW-F5 was further confirmed by the extensive reduction in the expression of MMP-9 in tumours harvested from mice treated with DW-F5 (Fig. 6c) confirming the *in vitro* results (Fig. 3l).

**Figure 7:**
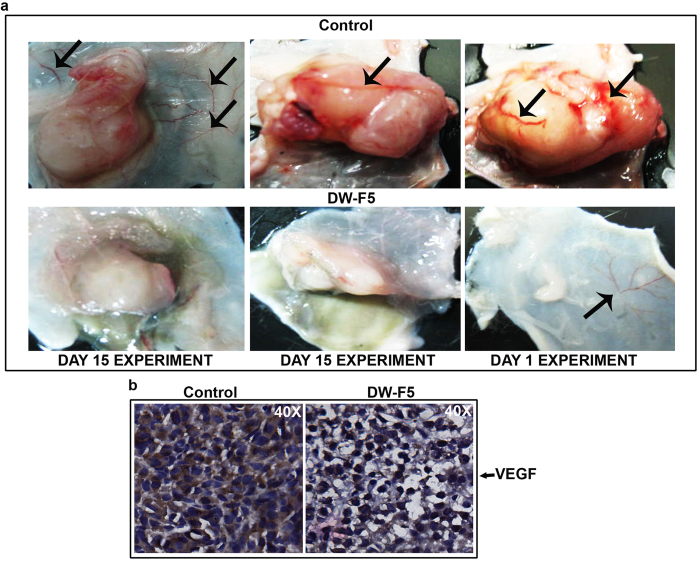
DW-F5 reduces angiogenesis in NOD SCID mice. **(a)** Images displaying the inhibition of blood vessel formation in and around the DW-F5 treated tumour mass with respect to untreated tumour. **(b)** Reduction of VEGF expression in tumour tissues of DW-F5 administered mice as assessed by IHC.

DW-F5 treatment markedly blocked blood vessel formation in and around the tumour mass (Fig. 7a). This observation was further supported by a considerable reduction in the major angiogenesis marker protein, VEGF in tumour sections of DW-F5-treated mice as assessed by IHC (Fig. 7b), corroborating the *in vitro* results (Fig. 3k). Taken together, the above noted novel observations strongly attest the candidature of DW-F5 as a potential nominee to be evaluated for its anticancer, anti-metastatic and anti-angiogenic efficacy against malignant melanoma.

### DW-F5 on further fractionation yields tryptanthrin and an aliphatic fraction, both of which are cytotoxic to melanoma cells

**Figure 8:**
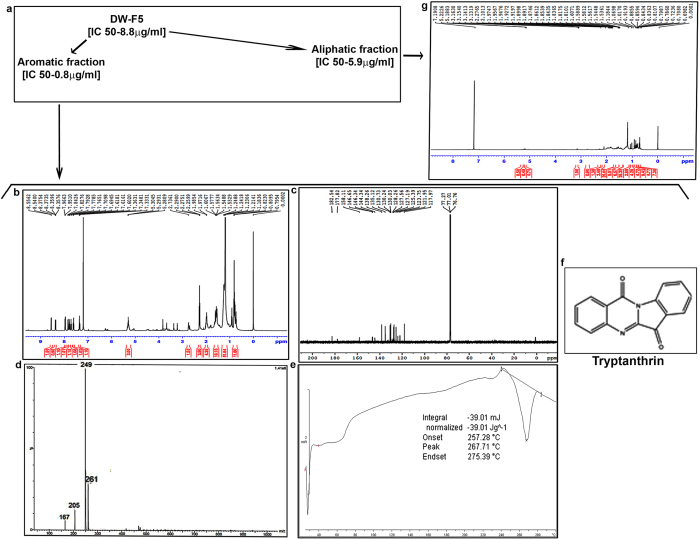
Purification of DW-F5 leads to the isolation of Tryptanthrin and another aliphatic fraction, which are cytotoxic to melanoma cells. (**a**) Flow chart depicting the purification of DW-F5 (**b–e**) ^1^H NMR, ^13^C NMR, Mass spectra and Melting point analysis of the aromatic fraction of DW-F5 (**f**) Chemical structure of tryptanthrin. (**g**) ^1^H NMR spectrum of the aliphatic fraction of DW-F5.

We tried to further purify DW-F5 by repeated column chromatography, which lead to the isolation of two cytotoxic components, one of which is aromatic and the other is aliphatic. While DW-F5 showed an IC 50 of 8.8 μg/ml, the aromatic fraction and the aliphatic fraction exhibited a substantial decrease in IC 50 values [0.8 and 5.9 μg/ml respectively] (Fig. 8a).

In the ^1^H NMR [Nuclear Magnetic Resonance] spectrum of the aromatic component, the peaks were observed in the region ranging from δ 7.33–8.55 ppm, confirming its aromatic nature and the peaks corresponds to the eight protons of the phenyl ring (Fig. 8b). The ^13^C NMR spectrum exhibited peaks in the region 117.97–182.54 corresponding to fifteen carbons of which 182.54 and 177.82 are characteristic of carbonyl carbons (Fig. 8c). In the ESI [Electrospray Ionization] mass spectrum, the molecular ion [M+H]+ was observed at m/z 249 (Fig. 8d). The melting point of the isolated compound was observed between 259–260 °C (Fig. 8e). Based on the above analytical data the isolated compound was identified as Indolo[2,1-b]quinazoline-6,12-dione [tryptanthrin] (Fig. 8f) (SI methods). The melting point and spectral data of the isolated compound is in accordance with the reported synthetic compound^18^.

In the ^1^H NMR spectrum of the aliphatic component, peaks are observed in the region, δ 0.8–5.3, which corresponds to protons in aliphatic compound/s (Fig. 8g). Silica gel column chromatography and spectral analyses is going on to further purify and identify the active molecule/s present in this fraction.

## Discussion

Despite intense research, the chemotherapeutic armamentarium for the treatment of malignant melanoma, one of the most aggressive forms of human cancer, remains limited^19^. The present study introduces DW-F5, a semi purified fraction isolated from the leaves of an indigenous plant, *Wrightia tinctoria*, as a potential formulation against melanoma. To the best of our knowledge, no information regarding the anti-tumour activity of this plant subsists in literature, except a report on the anti-inflammatory activity of its leaf extract^20^.

The efficacy of DW-F5 as an apoptosis inducer deserves prime mentioning in this context. Apoptosis is an energy-dependent cascade of molecular events characterized by membrane flip flop, caspase activation and subsequent cleavage of functional enzymes such as PARP leading to the systematic dismantling of cells^21^. In concordance with the currently acceptable dogma of apoptosis, DWF-5 induces such signature molecular events of apoptosis in melanoma cells demonstrating its chemotherapeutic potential.

From the experimental results, it is quite logical to presume that the reason behind the efficacy of DW-F5 to inhibit melanoma cells, resides in its ability to down-regulate the expression of some of the key molecules involved in melanoma progression. The pronounced ability of DW-F5 to inhibit MITF-M is a case in point. MITF-M is a transcription factor, which is considered as a melanoma oncogene and the master regulator of melanocyte development^22^. It is known that the ectopic co-expression of MITF and mutant BRAF leads to transformation of primary melanocytes^23^ while the abrogation of MITF-M activity in BRAF V600E MITF-M melanoma leads to dramatic tumour regression^24^. Besides this, disruption of the canonical Wnt pathway also abrogates growth of melanoma cells^25^, and constitutive over-expression of MITF-M rescues the growth suppression, which establishes the critical role of both β-catenin and MITF-M in melanoma development^15^. Phosphorylation status of ERK 1/2 has strong correlation to the malignant potential and progression of melanoma^26^. It has been shown that oncogenic BRAF induces phosphorylation of ERK 1/2^27^ leading to up-regulation of the transcription factor, Brn-2 and subsequently the up-regulation of MITF-M^22^. Up-regulation of β-catenin too induces Brn-2^28^ and hence leads to up-regulation of MITF-M^22^.

However, our data regarding the regulatory effect of MITF-M in DW-F5-induced cytotoxicity in multiple skin cancer cell lines indicate that the complete abolition of MITF expression, but not the blockage of Brn-2 expression, is the crucial factor for DW- F5-induced cell death. While a moderate reduction of MITF-M and a complete abolition of Brn-2 by 10 μg/ml of DW-F5 are not enough to induce 50% cytotoxicity in SK-MEL-2 cell line (IC50- 24.02 μg/ml), the same concentration, which caused complete abolition of MITF-M and Brn-2 expression induced more than 50% cytotoxicity in A375 (IC50- 9.8 μg/ml) indicating that MITF-M has an upper hand over Brn-2 in regulating DW-F5-induced cytotoxicity. Curiously enough, we could find that A431 (a non melanoma cell line) having comparatively low or nil expression of Brn-2 and MITF, and perhaps not depending on these molecules for its survival, was relatively non-sensitive to DW-F5 (IC50- 47.1 μg/ml).

About 50% of melanoma cells harbour mutation in BRAF kinase protein, which will lead to the deregulated activation of its downstream ERK/ MEK effectors. Given the significance of BRAF activation in melanoma cells, the down-regulation of BRAF in the DW-F5-treated cells and xenograft tissues assumes importance. Melanoma cells with BRAF mutation, through their elevated ERK1/2 signaling, activate NF-κB^29^, which in turn may lead to the transcription of proteins regulating melanoma progression and metastasis. Consideration of COX-2, a molecule downstream of NF-κB^30^, as an attractive target for melanoma treatment underscores the importance of NF-κB signaling axis in melanoma cells. An elevated Akt activation, which is reported in **~**77% of melanomas^31^ could also be a factor contributing to the elevated NF-κB signaling^32^,^29^. Interestingly, there are reports placing MITF-M and one of its direct targets, Bcl-2^33^,^34^, downstream of Akt- NF-κB signaling axis in melanoma cells^35^. The efficacy of DW-F5 to disarray the above-mentioned signaling axis operating in melanoma cells is demonstrated in its ability to down-regulate constitutive as well as induced activation of ERK1/2, Akt, NF-κB, COX-2 and Bcl-2.

The histopathology results of the liver cryosections and the normal range of the serum biochemistry profile of the animals administered with DW-F5, obtained from the toxicity studies confirms its pharmacological safety. Moreover, the non-toxic nature and the medicinal property of the leaves of *Wrightia tinctoria* is already established as its decoction is being used orally against jaundice, fever and tooth ache and also applied as poultice against psoriasis, mumps and herpes^1^.

Taken together, we observed that DW-F5 is inhibiting the expression of molecules such as BRAF, β-catenin, Akt and NF-κB, all of which are known to mediate signals converging in MITF-M, the master regulator of melanomagenesis. Hence the probable mechanism of the anti-tumour efficacy of DW-F5 is the down-regulation of BRAF, which leads to dephosphorylation of ERK1/2, along with the inhibition of β-catenin and Akt-NF-κB, together contributing to the inhibition of MITF-M, which in turn inhibits the anti-apoptotic molecule Bcl-2, ultimately leading to cell annihilation. However, more studies are needed to validate and confirm this assumption.

A considerable reduction in the vasculature in and around the treated tumour mass and the substantial down-regulation of VEGF expression in both *in vitro* and IHC results, illustrates the anti-angiogenic potential of DW-F5, an important event involved in the progression and spread of tumour. In harmony with the *in vitro* results, the substantial decrease observed in the expression of MMP-9, a major metastatic marker protein, in tissue sections of DW-F5 treated mice, confirms and attests the anti-metastatic potential of the fraction.

Spectral analyses indicates that DW-F5 is composed of an aromatic compound, tryptanthrin and an uncharacterized aliphatic fraction, both of which are cytotoxic to melanoma cells. Even though no study has been conducted on the anticancer effect of tryptanthrin against malignant melanoma, there are reports indicating its efficacy against multi drug resistant breast cancer cells^36^, colorectal adenocarcinoma^37^ and leukemia^38^,^39^. The cancer chemopreventive property of tryptanthrin has also been reported^40^ against intestinal tumours. Further studies are in progress to identify the aliphatic component of DW-F5. Investigation is also going on to identify the multiple molecular pathways regulating the anticancer activity of DW-F5, which may help to bring up the formulation as a potential drug for treating melanoma.

## Methods

### Plant materials

*Wrightia tinctoria* leaves were collected from Sanjeevani Naturopathic Centre, and authenticated by Dr. Reni Muringatheri (SI).

### Extraction, isolation and characterization of DW-F5

Successive extraction was done using solvents of ascending polarity and concentrated using a rotary evaporator (SI Methods). The DCM extract was subjected to silica gel column chromatography to isolate the semi purified fraction DW-F5 (SI Methods). The IR and ^1^H NMR spectra of the fraction were recorded for its characterization.

### Cell lines

FS was a gift from Dr. Bharat B. Agarwal. HEMa-LP was purchased from Gibco. All the other cell lines were procured from NCCS (SI).

### Antibodies and reagents

Antibodies against Caspases, p-ERK1/2, cleaved PARP and p-Akt were obtained from Cell Signaling (Beverly, MA, USA); those against Brn-2 and MITF-M were purchased from Abcam (Cambridge, UK). Antibodies against β-catenin, p-p65, Bcl-2, COX-2, PARP, VEGF and BRAF were purchased from Santa Cruz Biotechnology (Santa Cruz, CA, USA). Chemicals for purification were obtained from Merck and other chemicals were purchased from Sigma (SI).

### MTT assay

The cytotoxic effect of the extracts and fractions were determined by MTT assay as described earlier^41^ (SI Methods).

### Annexin V-Propidium Iodide staining and FACS analysis

The cells were treated with DW-F5 for 24 h, stained with annexin and PI and documented in a confocal microscope. The cells were treated with DW-F5 for 48 h, stained with annexin and PI and analysed using flow cytometry [SI Methods].

### Clonogenic assay

Clonogenic assay was performed in melanoma cells treated with or without DW-F5 as described previously^42^ (SI Methods).

### Western blot analysis

Total protein isolated from cells after indicated treatments were subjected to Western blotting as described earlier^41^ (SI Methods).

### DNA Fragmentation Assay

DNA Fragmentation Assay was done as described in SI Methods.

### Preparation of nuclear extracts and EMSA

EMSA was performed to evaluate DNA-binding activity of NF-κB as described earlier^42^ (SI Methods).

### Plasmid

The renilla luciferase expression plasmid, pKT2/mCa-Rluc-IRES-Puro was constructed as described earlier^43^ (SI Methods).

### Transfection

A375-Ren-Luc cells were generated by stably transfecting A375 cells with pKT2/mCa-Rluc-IRES-Puro vector using Lipofectamine 2000 (Invitrogen), according to manufactures protocol (SI Methods).

### Luciferase assay

The luminescence of A375-Ren-Luc cells in different tissues of NOD-SCID mice were analysed using the Dual-Luciferase Reporter Assay System (Promega) according to manufacturer’s protocol (SI Methods).

### *In vivo* studies

Animal studies were performed in accordance with the approved guidelines of Institute Animal Ethics Committee [IAEC] of Rajiv Gandhi Centre for Biotechnology, Thiruvananthapuram, Kerala, India. The anticancer, anti-metastatic and anti-angiogenic properties were evaluated using orthotopic model in NOD-SCID mice (SI Methods). The toxicological analyses was performed in Swiss albino mice. DW-F5 was administered in liposome-encapsulated form^44^ (SI Methods).

### Orthotopic model

Mice were injected with A375-Ren-Luc cells intradermally. DW-F5 was administered twice weekly for four weeks adopting two different types of drug treatment strategies and routes of administration (SI Methods).

### Metastasis model

Mice were injected with A375-Ren-Luc cells as described in the orthotopic model. Drug was administered twice weekly for eight weeks. The liver and lung tissues were analysed for metastatic spread using *in vitro* luciferase assay (SI Methods).

### Angiogenesis experiment

The anti-angiogenic potential was assessed by Western blot in melanoma cells and by observing the vascularisation on and around the tumour and the expression of VEGF in the tumour cryosections by using immunohistochemistry (IHC) analysis.

### Toxicological analyses

#### Acute toxicity

Doses of 0, 120 and 600 mg/Kg of DW-F5 were given to groups of six mice each^9^. Animals were euthanized on day 8. The liver tissue was analysed by histopathology using H&E staining^45^ and the serum was used to perform Liver Function Test (LFT) (SI Methods).

#### Chronic Toxicity

Doses of 0 and 120 mg/Kg of DW-F5 were given to groups of six mice each^9^. Animals were euthanized after 90 days and toxicity was measured as described above.

### Histology and Immunohistochemistry

The tumour tissues from mice were fixed and cryosectioned (SI Methods). Expression of different proteins in the tissue sections was assessed using Super Sensitive Polymer-HRP IHC Detection System (Biogenex, USA) (SI Methods).

### Isolation of tryptanthrin and aliphatic fraction form DW-F5

DW-F5 was subjected to silica gel column chromatography to isolate tryptanthrin and the aliphatic fraction (SI methods).

### Structure elucidation of bioactive compound

The pure compound was characterized by its ^1^H NMR, ^13^C NMR, ESI-MS spectra and melting point analyses (SI Methods).

### Spectral data

Indolo[2,1-b]quinazoline-6,12-dione (Tryptanthrin): Yellow solid; mp 259–260 °C (Lit. mp 258–260 °C, 18; ^1^H NMR (CDCl_3_, 500 MHz) δ 7. 34 (d, J = 7.9 Hz, ^1^H), 7.69 (dt, J = 7.9 and 2.1 Hz, ^1^H), 7.72 (dt, J = 7.9 and 2.1 Hz, ^1^H), 7.78 (dt, J = 7.9 and 2.1 Hz, ^1^H), 7.83 (d, J = 7.9 Hz, ^1^H), 7.95 (d, J = 7.9 Hz, ^1^H), 8.36 (dd, J = 7.9 and 2.1 Hz, ^1^H), 8.55 (d, J = 7.9 Hz, ^1^H); ^13^C NMR (CDCl_3_, 100 MHz) δ 117.9, 121.9, 123.7, 125.3, 127.1, 127.5, 130.2, 130.7, 135.1, 138.2, 144.3, 146.3, 146.6, 158.1, 177.8, 182.5; ESIMS (m/z) 249 [M+H]+.

### Statistics

The error bars represent ± S.D., taken from three independent experiments. Statistical significance was analysed by Student’s two-tailed, unpaired t test or two-way ANOVA followed by Tukey’s post test. Significance level was set at P < 0.05.

## Additional Information

**How to cite this article**: Antony, J. *et al.* DW-F5: A novel formulation against malignant melanoma from Wrightia tinctoria. *Sci. Rep.* **5**, 11107; doi: 10.1038/srep11107 (2015).

## Supplementary Material

Supplementary Information

Supplementary Information
