# Supplementary material for: DW-F5: A novel formulation against malignant melanoma from Wrightia tinctoria
Source: Sci Rep. 2015 Jun 10;5:11107. doi: 10.1038/srep11107 (PMC4650611; doi:10.1038/srep11107)

Supplementary information (SI)

DW-F5: A novel formulation against malignant melanoma from *Wrightia tinctoria*

Jayesh Antony[1#], Minakshi Saikia[1#], Vinod.V[1], Lekshmi. R. Nath[1], Mohana Rao Katiki[2], M.S.R. Murty[2], Anju Paul[1], Shabna A[1], Harsha Chandran[1], Sophia Margaret Joseph[1], Nishanth Kumar. S[3], Elizabeth Jayex Panakkal[1], Sriramya I.V[1], Sridivya I.V[1], Sophia Ran[4], Sankar S[5], Easwary Rajan[6] and Ruby John Anto[1*]

Plant material

Wrightia tinctoria leaves were collected during the month of April-May from Sanjeevani Naturopathic Centre, Mundur, Thrissur, Kerala. The plant was identified and authenticated by Dr.Reni Muringatheri from the same hospital. Voucher specimen is deposited in our herbarium.

Cell lines

The cervical cancer cell line HeLa, breast cancer cell line MCF-7, lung cancer cell line A549, colon cancer cell line SW480 and skin cancer cell lines, A375 (malignant melanoma), SK-MEL-2 (malignant melanoma) and A431 (squamous cell carcinoma) were procured from National Centre for Cell Sciences (NCCS, Pune, India) and the normal skin fibroblast cells, FS (Foreskin) was a kind gift from Dr. Bharat Agarwal, MD Anderson Cancer Center, Houston, USA and the normal skin epithelial cells, HEMa-LP (Human Epidermal Melanocytes, adult, lightly pigmented donor) was purchased from Invitrogen (Carlsbad, California, USA).

Reagents and chemicals

Dulbecco's modified Eagle's medium was obtained from Life Technologies (Grand Island, NY, USA). All the chemicals used for extraction, thin layer chromatography (TLC) and column chromatography were of analytical grade and were obtained from Merck Ltd, Mumbai, India. Silica gel (60-120 & 230–400 mesh) used for column chromatography and precoated silica gel 60 GF254 plates used for thin-layer chromatography (TLC) were purchased from Merck Ltd, Germany. All other chemicals were purchased from Sigma Chemicals (St. Louis, MO, USA). DAPI and Annexin V apoptosis detection kit were purchased from Santa Cruz Biotechnology (Santa Cruz, CA, USA).

Extraction and isolation of the semi purified fraction

The shade dried leaves of the plant were extracted successively using solvents of ascending polarity, i.e., hexane [HEX- 0.0], dichloromethane [DCM- 3.4] , ethyl acetate [EA- 4.4] and methanol [MET- 6.7], at 37°C in dark for 24h using a gyrorotatory shaker (150rev/ min). The extracts were filtered using Whatman Grade No.1 filter paper and concentrated at 40°C using a rotary flash evaporator (Buchi Rotavapor R-210).

The dichloromethane extract was loaded on a silica gel (60–120 mesh) column (450mm×30 mm) previously equilibrated with hexane and eluted successively with 100% hexane, linear gradient of hexane/dichloromethane (v/v, 95 : 5 to 5 : 95), 100% dichloromethane, linear gradient of dichloromethane/chloroform (v/v, 95 : 5 to 5 : 95), 100% chloroform and finally with 100% methanol. Eight fractions were collected from each combination. Among the four bioactive fractions collected from the first column, two most active fractions [Fr.15 & Fr.16] were combined and then further subjected to column (200mm×10 mm) chromatography with silica gel (230-400 mesh) as stationary phase. The column was eluted successively with 100% hexane, linear gradient of hexane/dichloromethane (v/v, 95: 5 to 0: 100), and finally

with 100% methanol. Sixteen fractions were collected from each combination, which were monitored using thin layer chromatography (TLC) and pooled together based on similar banding pattern to yield six fractions, out of which the fractions that showed highest activity and similar banding pattern [Fr.16-Fr.20] were pooled together and named as DW-F5, which was selected for all the *in vitro* and *in vivo* studies. The IR spectra (KBr) of the fraction was recorded on Thermo Nicolet Nexus 670 FTIR Spectrometer (ν in cm-1) and its ^{1}H NMR (500MHz) spectra was recorded on Bruker Avance 500 Nuclear Magnetic Resonance spectrometer taking the compounds in $CDCl_3$ using TMS as an internal standard. The chemical shifts (δ) were reported in parts per million (ppm) downfield from TMS.

MTT assay

The cytotoxic effect of DW-F5 was determined by MTT assay as described earlier [1] and the relative cell viability percentage is expressed as (Abs570 of treated wells/Abs570 of untreated wells) X 100. For all MTT assays, the cells were treated with the fraction for 72h. The DMSO concentration in all experiments, including control, was ≤ 0.2%.

Annexin V-Propidium Iodide staining

The cells were treated with DW-F5 for 24h and incubated in binding buffer containing fluorescein isothiocyanate (FITC) conjugated annexin V and Propidium Iodide at a concentration suggested by the manufacturer (Annexin V apoptosis detection kit, Santa Cruz Biotechnology) for a period of 15 min, followed by a PBS wash. The annexin V binding and Propidium Iodide uptake of cells were documented using confocal laser scanning microscope.

Annexin V-Propidium Iodide FACS analysis

Cells exposed to DW-F5 for 48h were fixed in 70% ice-cold ethanol. The fixed cells were then treated with RNase A (100mg/ml), annexin V and Propidium Iodide at a concentration

suggested by the manufacturer (Annexin V apoptosis detection kit, Santa Cruz Biotechnology) and subjected to flow cytometry using a flow cytometer (BD Biosciences).

DAPI staining

DAPI staining was used to analyze the apoptotic efficacy of DW-F5. A375 cells were grown on cover slips by placing them in 24 well plate and were treated with DW-F5 dissolved in DMSO for 24h. The cells were then washed with 1X PBS and were fixed using methanol-EDTA. The cells were stained with 1 µg mL^{-1} of DAPI and documented using a fluorescent microscope.

Western blot analysis

The expression and activation of survival signals such as ERK1/2, Akt, NF-κB, COX-2 and Bcl-2, were studied after pre-treating A375 cells with DW-F5 for different time intervals [0, 2, 4, 6, 8, 12, 24 & 48h respectively]. In all these cases PMA was used as an inducer [30 min treatment for NF-κB, ERK1/2 and Akt and 24h for COX-2 and Bcl-2]. Expression of molecules specific to melanoma signalling such as BRAF, Brn-2, β-catenin and MITF-M and the molecules involved in angiogenesis and metastasis including MMPs and VEGF were studied after treating the cells with DW-F5 for 12, 24 and 48h respectively.

Comment [JA1]: Reviewer #2
Comment 6: Legend in figure 2 should include an explanation about different times of incubation used in the different protein expression assays to help reader to understand easier all the information displayed.

Total protein isolated from cells after indicated treatments were subjected to Western blotting as described earlier[1]. Briefly, 60mg of whole cell protein was resolved on a 10–15% polyacrylamide gel, transferred to a PVDF membrane, incubated with the corresponding antibody and was detected by ECL (Millipore, Billerica, MA, USA).

DNA Fragmentation Assay

For assaying DNA fragmentation, cells were treated with DW-F5 for 48h, scraped into cold PBS and pelleted by centrifugation at 10,000 rpm for 2min. Pellet was dissolved in 400µl of

lysis solution (100mM NaCl, 5 mM EDTA, 10 mM Tris-HCl pH 8.0, 0.5% SDS) containing 0.5 mg/ml proteinase-K, followed by overnight incubation at 37°C. After incubation, 100μl of 5M NaCl was added and kept at 4°C for 12h followed by centrifugation at 13,000 rpm for 15 min. and DNA in the supernatant was extracted with 500μl of phenol: chloroform: isoamyl alcohol solution (25:24:1) followed by chloroform: isoamyl alcohol (24:1) treatment. DNA was precipitated with 1ml of ice-cold absolute ethanol, incubated at -70°C for 30min, centrifuged at 15,000 rpm for 5min, and air-dried. The pellet was dissolved in 20μl distilled water and incubated at 37°C for 30 min after adding 0.5μl of 10mg/ml RNase A. Equal concentrations of DNA were resolved on 2% agarose gel containing ethidium bromide. The bands were visualized under ultraviolet light and photographed using Flour S multiImager (Bio-Rad).

Clonogenic assay

Clonogenic assay was done as mentioned previously [2]. Briefly, 1×10^4 cells in six well plates were treated with DW-F5 for 72h and were replaced with fresh medium and incubated for 1 week. The clones developed were fixed in gluteraldehyde and stained using crystal violet. The clones were counted and compared with the control. Colony containing more than four cells was counted as one clone.

Preparation of nuclear extract and EMSA: EMSA was performed to evaluate DNA-binding activity of NF-κB as described earlier [2]. In brief, 10 μg of nuclear proteins was incubated for 30 min with ^{32}P-end-labeled double-stranded NF-κB oligonucleotide (5' TTGTTACAAGGGACTTTCC GCTG GGGACTTTCC AGGGAG GCGTGG-3') at 37 ^{o}C and the DNA protein complex was resolved in 6.6% non-denaturing polyacrylamide gel, which was dried and visualized by Phosphor Imager (Personal Molecular Imager FX, Bio-Rad).

Plasmid

The renilla luciferase (Rluc) sequence was isolated out of the pAC-hRluc (Addgene; Cambridge, MA, USA) plasmid and then ligated into the pKT2/mCa-IRES-Puro plasmid (Dr. Andrew Wilber; SIU-SOM, Springfield, IL, USA) between the CAGGS promoter and the IRES sequence. The final plasmid allows for renilla luciferase expression and puromycin selection to be driven under the CAG promoter [3].

Transfection

Cells were stably transfected with pKT2/mCa-Rluc-IRES-Puro vector and empty vector using Lipofectamine 2000 reagent according to manufactures protocol (Invitrogen, Carlsbad, California, USA). Briefly, the volume of DNA sample as well as the transfection reagent (at 1:1 ratio) were mixed in opti-MEM and added to 60-70% confluent cells and incubated for 5-7h followed by replacing opti-MEM with 10 % DMEM. The resulting transfected cells were later selected using puromycin (1µg/ml).

Luciferase assay

Xenograft generated in SCID mice using A375 Ren-Luc-transfected cells were analysed for luminescence using the Dual-Luciferase Reporter Assay System (Promega, Madison, WI, United States of America) as per the manufacturer's protocol. Briefly, 100 µl of the passive lysis buffer (1X) was added to the homogenised tissues obtained from the xenograft samples, mixed intermittently and finally passed through a 1ml syringe. The resulting lysate was centrifuged at 1500 rpm for 5min followed by the separation of 20 µl supernatant to which equal amount of LAR substrate was added and the luciferase activity was measured in a Luminometer (Promega Glomax 20/20). 20 µl of the stop and glow reagent was added to the above mixture and the relative luciferase activity was measured in the Luminometer

(Promega Glomax 20/20). The relative renila luciferase activity [Rluc], was normalized using the respective normal tissues.

In vivo studies

All *in vivo* experiments were approved by the Institute's animal ethical committee (IAEC).

Liposomal encapsulation of DW-F5 fraction

DW-F5 was encapsulated into the uni-lamellar liposome formulation containing phosphatidyl choline and cholesterol as per the method described earlier [4]. Briefly, 5 mg DW-F5, 45 mg phosphatidyl choline and 5.8 mg cholesterol were dissolved in 3:1 mixture of chloroform and methanol (100 ml). After dissolving completely, the solvent was removed using a vacuum rotary evaporator and the residue was suspended in sterile PBS. This solution was sonicated to ensure homogeneity and stored at 4°C. This preparation was administered to experimental animals at a dose of 120 mg/kg body weight. The control animals were injected with empty liposome vehicle. The drug treatment was continued for a period of four weeks after which the mice were euthanized in CO_2 chamber.

Orthotopic xenograft model

The xenograft studies to evaluate the efficacy of DW-F5 against human skin cancer [melanoma] were carried out in NOD-SCID (NOD.CB17-*Prkdc*^{scid}/J) mice. Male NOD SCID mice of six to eight weeks old were used in this study and were maintained in animal research facility of our institute. In the standard cage conditions (temperature between 19-25°C, relative humidity 30-70% and illumination cycle set to 12h light and 12h dark), animals were housed in the groups of 4 mice for experiments. Autoclaved rice husk was used as bedding material and autoclaved rodent feed and water was given to animals *ad libitum*. All animal handling and experimental procedures were carried out under pathogen-free conditions in laminar air flow.

A total of 6 groups of animals [eight per group] were included in the study. Animals in all groups were injected with 3 X 10^6 A375-Ren-Luc cells in 100 µl PBS intradermally on the lower hind flank. Two different prophylactic strategies have been used in this experimental context. In the first strategy [Day 15 experiment], DW-F5 was administered on well developed tumour, after 15 days of tumour cell implantation, while in the second strategy [Day 1 experiment], DW-F5 is administered starting from the next day of tumour implantation. In both these strategies, DW-F5 (120 mg/Kg/animal in 100 µl PBS) administration is done via two different routes including intradermal [on the tumour site] and intraperitoneal, twice weekly for a total period of four weeks. Group 1 and group 4 [4A and 4B] includes animals which are not receiving any treatment, Group 2 and 5 [5A and 5B] includes animals receiving the intradermal mode of drug administration and finally Group 3 and 6 [6A and 6B] includes animals receiving the intraperitoneal mode of drug administration. The tumour growth was monitored every two days and tumour volume was measured every week. Throughout the study, all cages were checked every day at regular intervals for any dead or moribund animals, in order to carry out immediate necropsy. As the study could not find any measurable tumour in the day 1 experiment groups [Group 5 and 6] after the completion of the experiment, a set of animals from each groups were again kept under observation [Group 5B and 6B] for two more weeks along with one set of their untreated animals [Group 4B].

Toxicological analysis

Animals were euthanized after the experimental period using CO_2. Liver tissues of the sacrificed animals was collected and fixed in 4% paraformaldehyde and preserved in 30% sucrose. Tissue Sections obtained from such samples were stained with haematoxylin and eosin [5]. Liver Function Test [LFT] from the serum samples of animas was conducted to analyse the serum levels of various parameters such as total protein, albumin, globulin (all in

g/dL) and bilirubin [Total and direct] (mg/dL). The activity of serum alkaline phosphatase (ALP), serum glutamate oxaloacetic transaminase (GOT) and glutamate pyruvic transaminase (GPT) were also estimated (DDRC Laboratories, Trivandrum) and compared with that of normal controls (http://en.aml-vet.com/animal-species/mouse/).

Sample collection and preparation of tissue cryosections

At the termination of the experiment, all the animals were sacrificed using CO_2. Tumour from both untreated and DW-F5 treated animals were excised, washed in ice-cold PBS and photographed. The radii of tumour in two different planes were measured using a vernier calliper and the volume was calculated as (length X width^2)/2.

Tissue for sectioning were excised and fixed in 4% paraformaldehyde solution overnight, transferred to 30% sucrose solution till it sinks. The sunken tissues were kept immersed in O.C.T. compound [Tissue-Tek] and allowed to freeze by keeping in the cryostat [Leica CM 1850 UV] set at -20 ^{o}C. Tissue sections of 7µm thickness were cut from the frozen tissue samples and were kept in -80 ^{o}C till histopathological or immunohistochemical analyses.

Histology and immunohistochemistry

For histopatholgical examination, the tissue sections were kept in room temperature for 1h, and subsequently passaged twice through PBS, then through distilled water for 5 min each and stained with hematoxylin for 30 min. Excess stain was washed off and the slides were dipped in differentiation solution (3 sec), tap water (10 min) and 70% Isopropyl alcohol (5 min) before counter stained with eosin solution for 1 min. The sections were kept in 100% Isopropyl alcohol for 2 min twice, cleared in xylene and mounted in DPX mountant. Stained sections were observed under a light microscope and photographed.

Immunolocalization of specific proteins in the tissue sections was done using the Super Sensitive Polymer-HRP IHC Detection System (Biogenex, USA). The tissue sections were kept in room temperature for 1h, and subsequently passaged twice through PBS and then

through distilled water for 5min each and subjected to heat-induced antigen retrieval in citrate buffer. Nonspecific antibody binding sites on tissue sections and endogenous peroxidase activity were blocked by appropriate reagents supplied with the kit. The primary antibody diluted in TBST was added to the tissue sections. After incubating for 2h at room temperature, the unbound antibody was washed off with PBS-T (Phosphate Buffered Saline with 0.1% Tween-20). The sections were then covered with secondary antibody provided in the kit and incubated for 20min at room temperature followed by rinsing it in PBS-T. Immunostaining was visualized using diaminobenzidine chromogen, counterstained with Mayer's hematoxylin and the sections were mounted using SuperMount® mounting medium. Photomicrographs were captured using a Nikon Eclipse microscope equipped with Image-Pro Plus software.

Fractionation of DW-F5

About 20mg of bioactive fraction (DW-F5) was further purified using silica gel (230-400 mesh) column (200mm×10mm). The column was eluted step wise with 5 ml of hexane, hexane: dichloromethane, methanol in various ratios. 41 fractions were collected and concentrated on a rotary evaporator. An aliquot of all the concentrated fractions were loaded on the activated silica gel TLC plates (20 cm×20 cm). The plates were developed using hexane: dichloromethane (15:85, 20:80, 30:70, 17:83), methanol: chloroform (9:1) and methanol: chloroform: water (8:1:1). The spots were located by observing under UV light. Fractions having same number of spots (Fr.22, 23, 24 & 25 and Fr.26, 27, 28, 29 & 30) with similar Rf values on TLC plate were pooled. The pooled fractions were numbered (Fr.5& Fr.6) and were tested for cytotoxic activity. Among these, fraction number five (Fr.5) obtained from third step chromatography showed a single spot in TLC profile. This pure compound was subjected to various spectroscopic techniques for elucidation of the structure.

Fraction number 6, which contains a number of spots, was also subjected to spectral analyses in order to identify its composition.

Structure elucidation of bioactive compound and characterization of aliphatic fraction

The ^1H NMR (500 MHz) spectra and ^{13}C NMR (100 MHz) spectra were recorded on Bruker Avance 500 Nuclear Magnetic Resonance spectrometer taking the compounds in $CDCl_3$ using TMS as an internal standard. The chemical shifts (δ) were reported in parts per million (ppm) downfield from TMS. Mass measurements were carried out on CEC-21-110B double focusing mass spectrometer operating at 70 eV using direct inlet systems and are given in mass units (m/z) under Electron Spray Ionization conditions preparing sample solution in Methanol. The melting point of the pure compound was measured using a differential scanning calorimeter (DSC) with a Mettler Toledo DSC 822e instrument (Mettler-Toledo, Schwerzenbach, Switzerland). Temperature ranges from 30 to 300 °C was employed.

References

1 Bava, S. V. *et al.* Sensitization of taxol-induced apoptosis by curcumin involves down-regulation of nuclear factor-kappaB and the serine/threonine kinase Akt and is independent of tubulin polymerization. *J. Biol. Chem.* **280**, 6301-6308, doi:M410647200 [pii]10.1074/jbc.M410647200 (2005).

2 Puliyappadamba, V. T. *et al.* Nicotine-induced survival signaling in lung cancer cells is dependent on their p53 status while its down-regulation by curcumin is independent. *Mol. Cancer.* **9**, 220, doi:10.1186/1476-4598-9-2201476-4598-9-220 [pii] (2010).

3 Volk-Draper, L. D., Rajput, S., Hall, K. L., Wilber, A. & Rana, S. Novel Model for Basaloid Triple-negative Breast Cancer: Behavior *In Vivo* and Response to Therapy. *Neoplasia* **14**, 926-942 (2012).

4 Ruby, A., Kuttan, G., Dinesh Babu, K., Rajasekharan, K. & Kuttan, R. Anti-tumour and antioxidant activity of natural curcuminoids. *Cancer. Lett.* **94**, 79-83 (1995).

5 Prasad, S. B. & Giri, A. Antitumor effect of cisplatin against murine ascites Dalton's lymphoma. *Indian J. Exp. Biol.* **32**, 155-162 (1994).

Figure Legends

Supplementary Figure 1. Cytotoxic effect of different organic extracts of *Wrightia tinctoria* in human cancer cell lines of different origins. (a-d) Effect of hexane, dichloromethane, ethyl acetate and methanol extracts on various human cancer cell lines. A total of 3000 cells in triplicates were exposed to the indicated concentrations of the above mentioned extracts for 72h and subjected to 3-(4,5 dimethylthiazol-2-yl)-2,5-diphenyltetrazolium bromide (MTT) assay. Relative cell viability was determined as percentage absorbance of treated cells over untreated control. Data represent three independent sets of experiments. The error bars represent ± S.D. P- value was analyzed by Student's t test or ANOVA, in which * (P≤ 0.05), ** (P≤ 0.01), and # (P≤ 0.05) represent statistical significance.

Comment [JA2]: Reviewer 3 Comment 4: The statistical analysis was not appropriately used in the quantification. Please go through the manuscript and provide the statistic analysis in all quantification data.

Supplementary Figure 2. Cytotoxic effect of the dichloromethane extract of *Wrightia tinctoria* in melanoma and non-melanoma skin cancer cell lines. Effect of dichloromethane extract on human melanoma cell lines (A375 and SK-MEL-2) and the non-melanoma cell line (A431). A total of 3000 cells in triplicates were exposed to the indicated concentrations of the extract for 72h and subjected to 3-(4,5 dimethylthiazol-2-yl)-2,5-diphenyltetrazolium bromide (MTT) assay. Relative cell viability was determined as percentage absorbance of treated cells over untreated control. Data represent three independent sets of experiments. The error bars represent ± S.D. P- value was analyzed by Student's t test or ANOVA, in which * (P≤ 0.05), ** (P≤ 0.01), and # (P≤ 0.05) represent statistical significance.

Comment [JA3]: Reviewer 3 Comment 4: The statistical analysis was not appropriately used in the quantification. Please go through the manuscript and provide the statistic analysis in all quantification data.

Supplementary Figure 3. TLC chromatogram of the dichloromethane extracts and the purified column fractions. Representative TLC pictures of the crude and semi-purified fractions obtained after successive silica gel column chromatography, viewed under long & short UV and visible light, respectively. Decrease in IC50 values in A375 cells treated with the semi-purified fractions (as indicated in the bottom panel) indicates increase in purity.

Supplementary Figure 4. Spectroscopic analyses of the isolated active fraction, DW-F5

(a). IR spectra (KBr) were recorded on Thermo Nicolet Nexus 670 FT-IR Spectrometer. A region from 500 to 4000 cm^{-1} was used for scanning. (b) The ^1H NMR (500 MHz) spectra was recorded on Bruker Avance 500 Nuclear Magnetic Resonance spectrometer taking the fraction in $CDCl_3$ using TMS as an internal standard. The chemical shifts (δ) were reported in parts per million (ppm) downfield from TMS. A region from 0 to 10ppm for ^1H was employed for scanning. About 7.5 mg of isolated fraction was used for recording the spectra.

Supplementary Figure 5. DW-F5 induces nuclear condensation and inhibits the clonogenic potential of A375 cells

(a) DAPI staining of DW-F5 treated A375 cells shows condensed chromatin (apoptotic condition) and exhibits bright fluorescence compared to the normal nuclei in the untreated control. **(b,c)** Viability of melanoma cells treated with or without DW-F5 as determined by clonogenic assay. Statistical significance was analyzed by Student's t test. ** $P \leq 0.01$; # $P \leq 0.001$.

Supplementary Figure 6. Comparison of expression pattern of Brn-2 and MITF-M in different skin cancer cell lines [melanoma & non-melaoma]

(a) Effect of DW-F5 on human melanoma cell lines (A375 and SK-MEL-2) and the non-melanoma cell line (A431). A total of 3000 cells in triplicates were exposed to the indicated concentrations of the extract for 72 h and subjected to 3-(4,5 dimethylthiazol-2-yl)-2,5-diphenyltetrazolium bromide (MTT) assay. Relative cell viability was determined as percentage absorbance of treated cells over untreated control. Data represent three independent sets of experiments. The error bars represent ± S.D. Statistical significance was analyzed by ANOVA, * $P \leq 0.05$; ** $P \leq 0.01$; # $P \leq 0.001$; ns non significance (b) Western blotting was performed in DW-F5 treated melanoma [A375 & SK-MEL-2] and non-

Comment [JA4]: Reviewer #3 Comment 3: The DNA fragmentation assay did not clearly indicate the DNA fragmentation induced by DW-F5 because the untreated sample also exhibited degradation of DNA (figure 1h). Moreover, figure 1c-1g used many apoptotic markers, the authors should elucidate these results for readers to comprehend the results.

Response: Figure 1h is now changed to Figure 2i and 1c-1g is changed to 2c-2h. We did not get any fragmentation in the control sample, except a small smear, which appeared when we tried to make the fragments in the treated wells clearer. Moreover, we have also conducted DAPI staining, Annexin V/PI staining and Annexin PI/ FACS analyses to confirm induction of apoptosis by DW-F5 (Please see Fig 2a & b and Supplimentary Fig 5a). The results have been discussed briefly in the modified version.

Comment [JA5]: Reviewer 3 Comment 1 : The statistic analysis was missed in many quantification data. For figure 1b, what ** and *** mean? No description in the legends. How authors compared and determined the significance of difference among different treatments in time-course and dose dependent manners were not stated (Figure 3b, 3e-3j, Figure 4a and 4b).

Response: Figure 1b has been changed to Supplementary Figure 5c and 3 has been changed to 1. We have done the statistical analysis and included it in the figures and in respective legends.

Comment [JA6]: Reviewer 3 Comment 4: The statistical analysis was not appropriately used in the quantification. Please go through the manuscript and provide the statistic analysis in all quantification data.

melanoma [A431] cells to compare the expression pattern of two key melanoma specific molecules.

Comment [JA7]: Reviewer 3
Comment 2: The authors used several skin cancer cell lines and finally found that only A375 cells responded apparently to DW-F5. It will be of interest to compare the signaling molecules in figure 2 between/among these cells lines (at least pick one) after DW-F5 treatment.

Supplementary Figure 7. A cartoon representation of the detailed experimental plan of orthotopic xenograft model.

Two drug treatment strategies were adopted to analyze the *in vivo* efficacy of DW-F5. In the first strategy [Day 15 experiment], DW-F5 administration was started after 15 days of tumour cell implantation [on well developed tumour], while in the second strategy [Day 1 experiment], DW-F5 is administered starting from the next day of tumour cell implantation. As there was no measurable tumour in the Day 1 experiment groups after the stipulated time of DW-F5 treatment [Group 5A and 6A], a set of animals from each group were again kept under observation [Group 5B and 6B] for two more weeks, without further DW-F5 treatment, along with one set of untreated animals [Group 4B].

Supplementary Figure 1

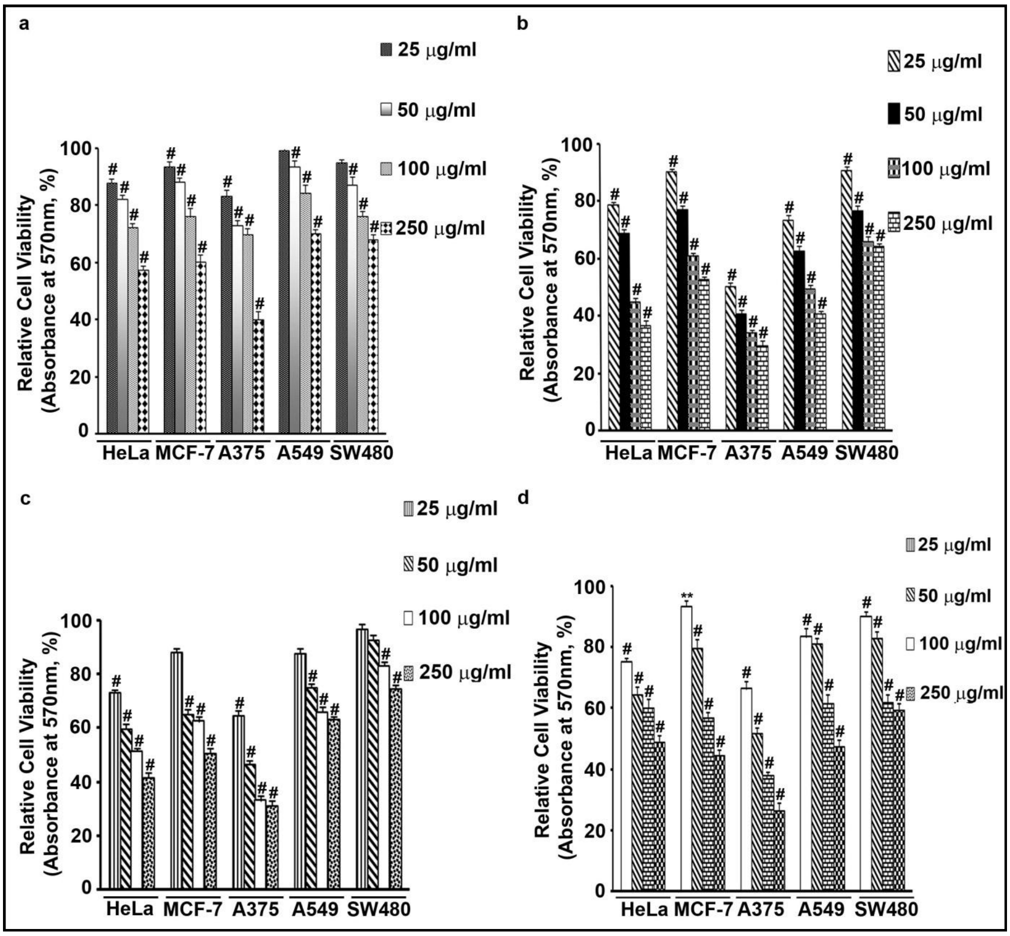

Supplementary Figure 2

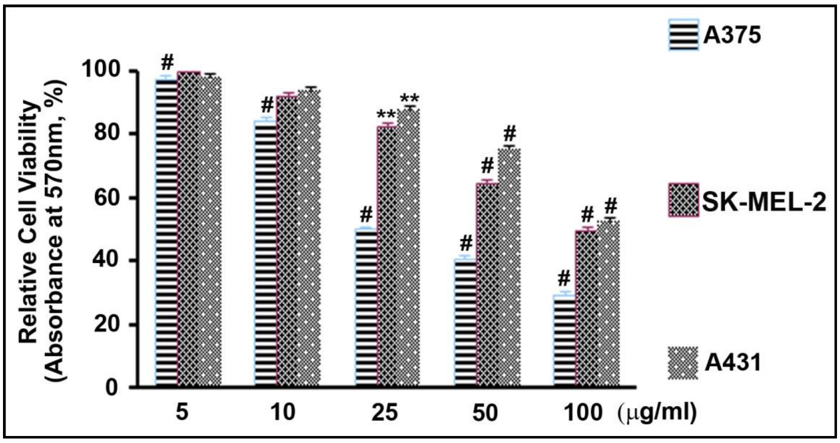

Supplementary Figure 3

	DCM EXTRACT		
Fractions	Crude	First column<br>Fr.15 & Fr.16	Second column<br>Fr.6- Fr.9
TLC chromatogram	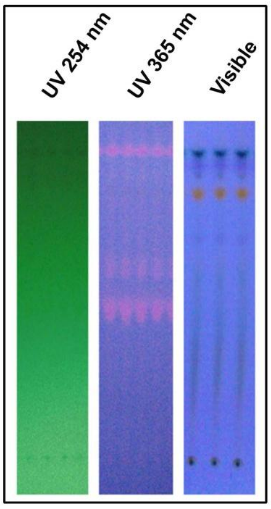	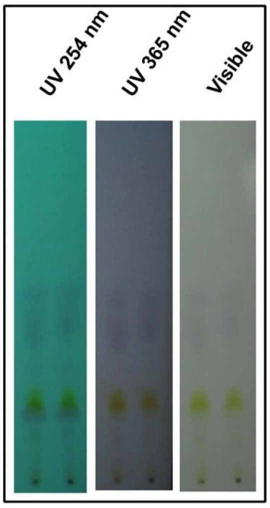	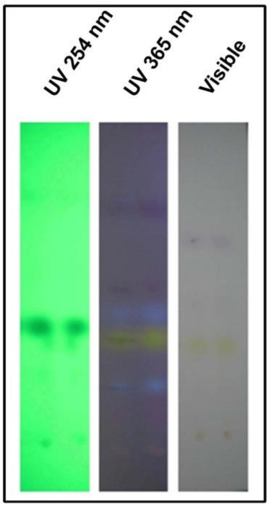
IC 50 (μg/ml)	20	9.23	8.8

Supplementary Figure 4

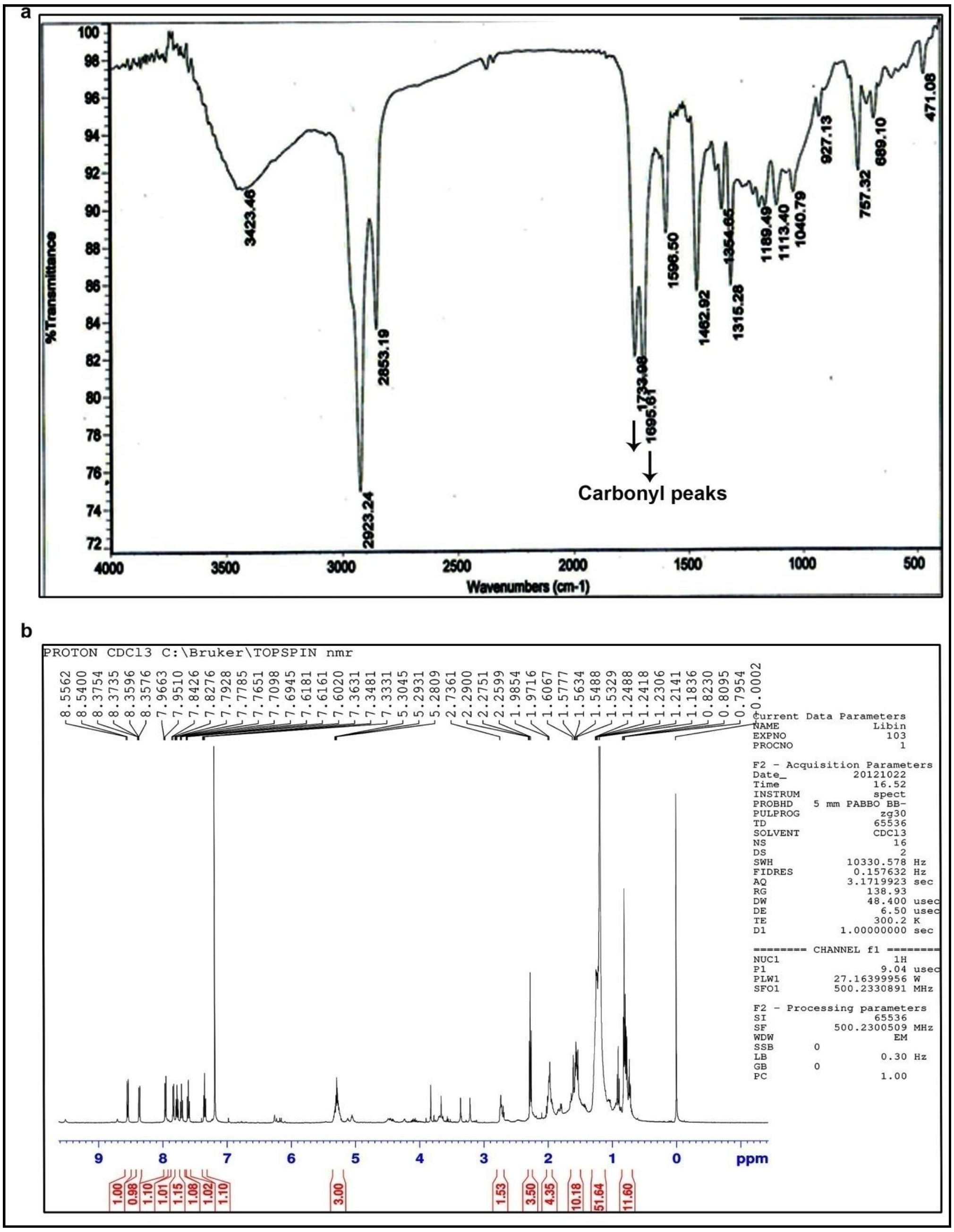

Supplementary Figure 5

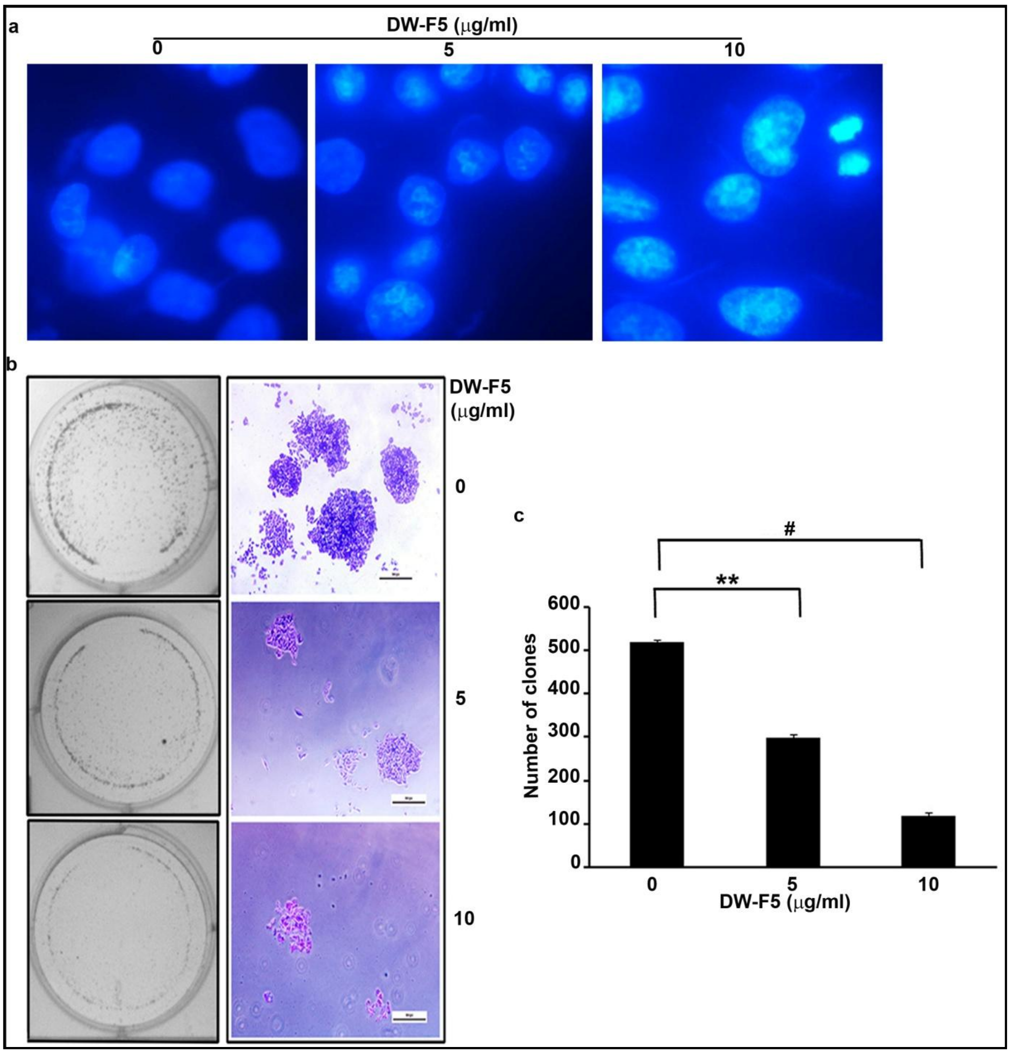

Supplementary Figure 6

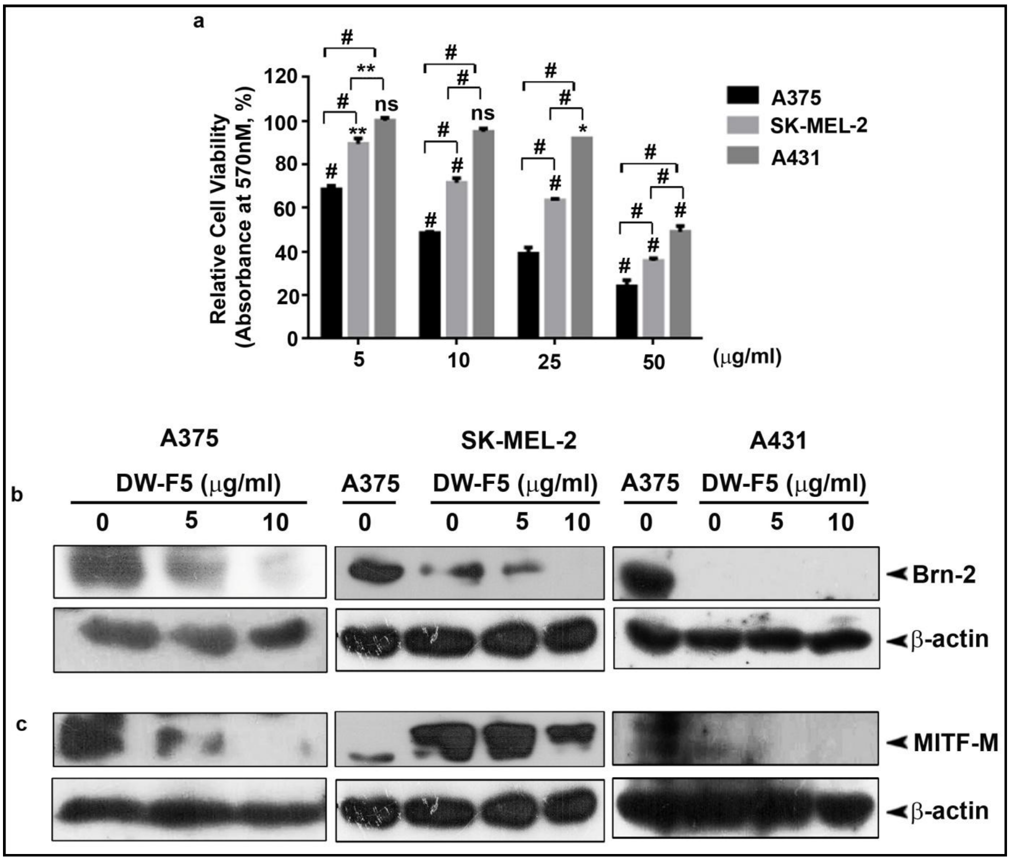

Supplementary Figure 7

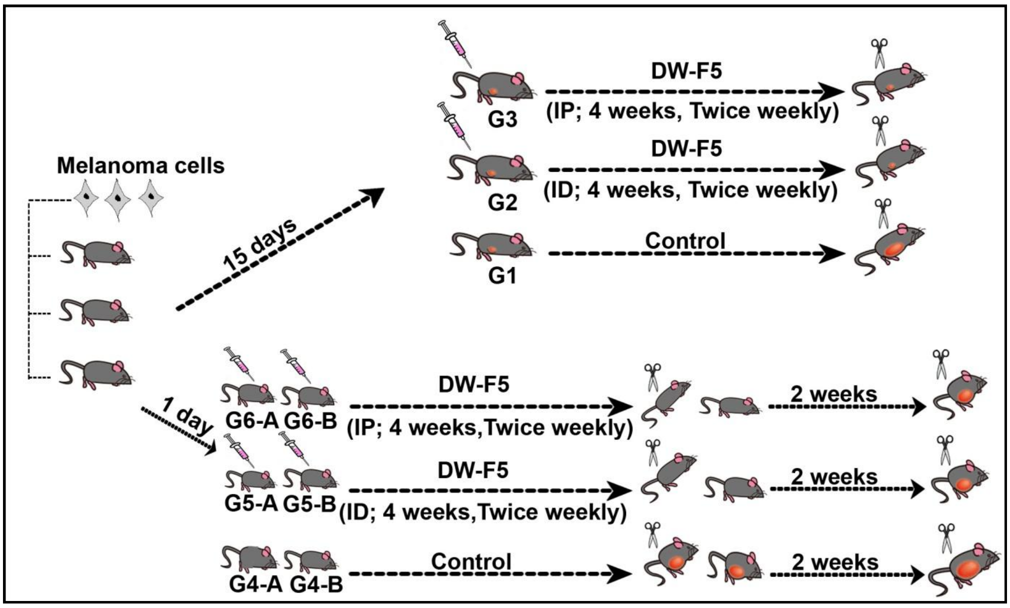

Supplement: Supplementary Information [file srep11107-s2.pdf]
